# Burden of Care Implications and Association of Intracranial Hypertension With Extremely Severe Post-traumatic Amnesia After Traumatic Brain Injury: A 5-Year Retrospective Longitudinal Study

**DOI:** 10.3389/fneur.2019.00034

**Published:** 2019-01-29

**Authors:** Nhung T. Quach, Reza Ehsanian, Benjamin Dirlikov, Samantha Sechrist, Jyodi Mohole, Stephen McKenna, Linda Isaac, Thao T. Duong

**Affiliations:** ^1^Rehabilitation Research Center, Santa Clara Valley Medical Center, San Jose, CA, United States; ^2^Department of Neurosurgery, Stanford University, Stanford, CA, United States; ^3^Physical Medicine and Rehabilitation, Santa Clara Valley Medical Center, San Jose, CA, United States; ^4^Department of Orthopedic Surgery, Stanford University, Stanford, CA, United States

**Keywords:** Traumatic brain injury (TBI), post-traumatic amnesia (PTA), burden of care, intracranial hypertension (ICH), FIM® instrument (FIM)

## Abstract

Post-traumatic amnesia (PTA) is characterized by a state of disorientation and confusion following traumatic brain injury (TBI). Few studies have looked at the effect of prolonged PTA on the functional outcomes beyond 1 year post-injury. This study aims to evaluate the burden of care in individuals with extremely severe PTA (esPTA; PTA >28 days) from acute inpatient rehabilitation admission to 5 years post-injury as well as the association between intracranial hypertension (ICH; Intracranial pressure (ICP) ≥20 mmHg) and esPTA status. Three hundred and forty-two individuals with moderate to severe TBI enrolled in the Northern California TBI Model System (TBIMS) of Care were included in this study. The FIM® instrument was chosen as the outcome measurement as it is a widely used functional assessment in the rehabilitation community. Repeated measure ANOVA revealed greater burden of care based on FIM® total scores (*p* < 0.001) from admission to 5-year follow-up for the esPTA group compared to the non-esPTA group (PTA ≤ 28 days). Unlike the non-esPTA group where FIM® total score plateaued 1 year post-injury, FIM® total score continued to improve up to 2 years post-injury for the esPTA group. The odds of developing esPTA was ~3 times higher for individuals with ICH vs. individuals without ICH (*p* < 0.001). In conclusion, individuals with esPTA have increased short- and long-term burden of care and the presence of ICH during hospitalization increased the odds of experiencing esPTA. These results may help the rehabilitation team and family in planning care post rehabilitation discharge.

## Introduction

Traumatic brain injury (TBI) is one of the leading causes of death and disability worldwide (1). According to the Centers for Disease Control and Prevention (CDC), in 2010 there were ~2.5 million emergency department visits, hospitalizations, and deaths due to TBI in the U.S. (2). The CDC estimated that ~16% of annual hospitalizations due to a traumatic injury were related to TBI (2). TBI accounts for substantial health care costs in the US; in 2010, there were 21.4 billion dollars in charges for TBI-related admissions as well as 8.2 billion dollars charged for ED visits (3). In addition to the financial impact, given the increased burden of care faced by caregivers of patients with TBI, it is not surprising that caregivers report being “overburdened with responsibilities” (4) and suffer from a high level of caregiver distress (5). These factors highlight the importance of TBI research to improve early diagnosis, prognosis, and treatment to maximize functional recovery and reduce the burden of care for individuals with TBI.

TBI can have a devastating impact ranging from impaired physical capability to altered cognition, which impedes functional independence (2). The cognitive sequelae following TBI often includes post-traumatic amnesia (PTA) characterized by disorientation, confusion, restlessness, inability to recall events, poor attention, and agitated behavior (6–9). In individuals with complicated mild TBI, Hart et al. found that shorter PTA duration (≤1 week) predicted greater cognitive performance and less disability at 6 months post-injury vs. patients with a PTA duration >1 week (10). Eastvold et al. used PTA duration as a predictor for outcomes after TBI and found that patients with PTA <30 days were more than three times as likely to be living independently 1 year after injury when compared to patients with PTA duration >30 days (11). Increased PTA duration has also been linked to decreased productivity at 1 year post-injury (12) as well as higher rates of severe disability and poor recovery 2 years post-injury (13). Asikainen et al. (14) found PTA duration over four weeks was associated with severe disability in adults as measured by the Glasgow outcome scale. These findings highlight that across different follow-up periods and different outcome metrics, longer PTA duration is invariably associated with worse outcomes, which increases the burden of care. There is a paucity of studies evaluating functional outcomes relating to PTA duration beyond 1 year post-injury, limiting long-term care planning. To guide long-term discharge planning, it would be beneficial to have more studies investigating the association of PTA duration on functional improvements and burden of care beyond 1 year post discharge.

In addition, while PTA duration is clinically used as a criterion to classify TBI severity (2, 15), the presence of PTA itself is an important outcome of TBI that is related to hospital length of stay (16) and affects patients' quality of life (17). Studies elucidating objective measures to help predict PTA duration would be of clinical utility for clinicians planning the rehabilitation and discharge of patients with TBI. One such study by Sherer et al. used age, years of education, year of injury, GCS, length of coma, pupillary responsiveness, and intracranial operations to predict length of PTA (18). Another potential measure that is also associated with poor outcomes after TBI is intracranial hypertension (ICH; Intracranial pressure (ICP) ≥20 mmHg) (19–21). Many patients with severe TBI experienced ICH and had ICP monitoring. ICP is an objective, accurate, and important factor in TBI management, and may be associated with PTA duration. However, few studies look at the association between PTA duration and ICH.

The primary aim of this study was to investigate if individuals with extremely severe PTA (esPTA; PTA >28 days) have long-term increased burden of care compared to non-esPTA (PTA ≤28 days) individuals. The FIM® instrument (FIM) was used as the main measurement because the FIM instrument is a widely used functional assessment in the rehabilitation community and can be interpreted into the required hours of care, which reflects the burden of care (22, 23). As a secondary analysis, this study investigates the association between esPTA and ICH. This study aims to investigate the differences in the burden of care for individuals with and without extremely severe PTA up to 5 years post-injury and the association of ICH with esPTA.

## Methods

### Participants

**Figure 1:**
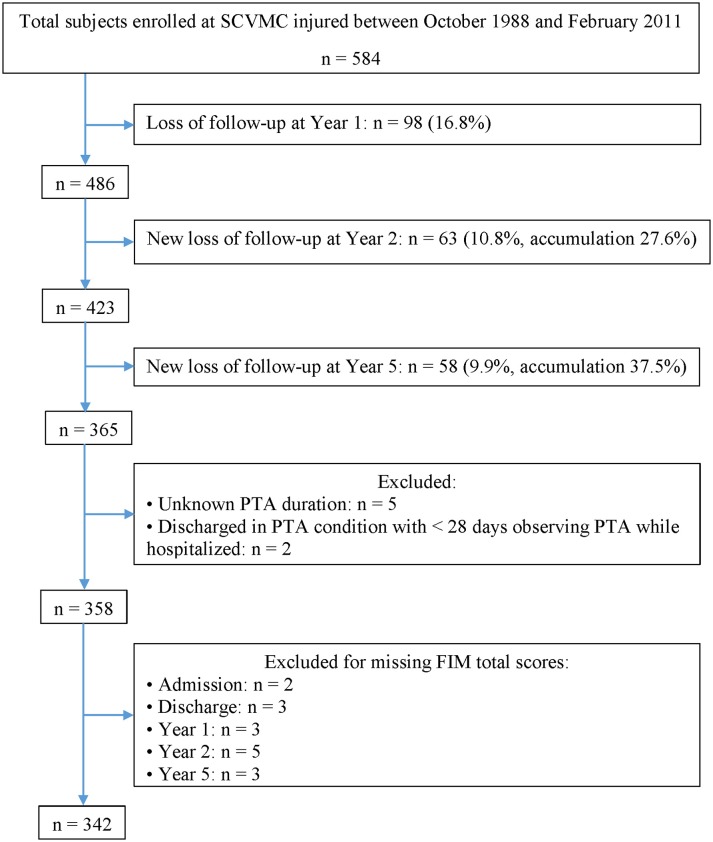
Flowchart of the study participants.

Individuals enrolled in the Northern California Traumatic Brain Injury Model System of Care (TBIMS) (24) longitudinal study who were injured between October 1988 and February 2011 and completed follow-up interviews up to 2016 were selected for this retrospective study. The inclusion criteria for TBIMS include English and/or Spanish-speaking patients with moderate to severe TBI (either having PTA >24 h, trauma related head computed tomography abnormalities, loss of consciousness >30 min, or GCS in the ER <13) who sustained an injury in California, presented to an acute care hospital within 72 h of injury, were at least 16 years old at the time of injury, and provided consent to participate in the TBIMS by patient, family, or guardian. A total of 584 participants were enrolled between October 1988 and February 2011, however, only 342 individuals (59%) with known PTA duration and FIM scores across all time points were included in the current study (Figure 1).

### Data Collection for Northern California Traumatic Brain Injury Model System of Care

Participants' medical information from acute hospitalization, such as ICP, and inpatient rehabilitation, such as FIM scores, was abstracted from medical records. Demographics and pre-injury history data were collected via participant interview during inpatient rehabilitation. Follow-up assessments, including FIM assessment, were conducted via phone interviews at one, two, and 5 years post-injury per TBIMS protocol (24). FIM scores obtained though phone interviews have been shown to reliable and have good intermodal agreement to in-person assessments (25, 26).

### Measures

#### FIM Instrument

**Figure 2:**
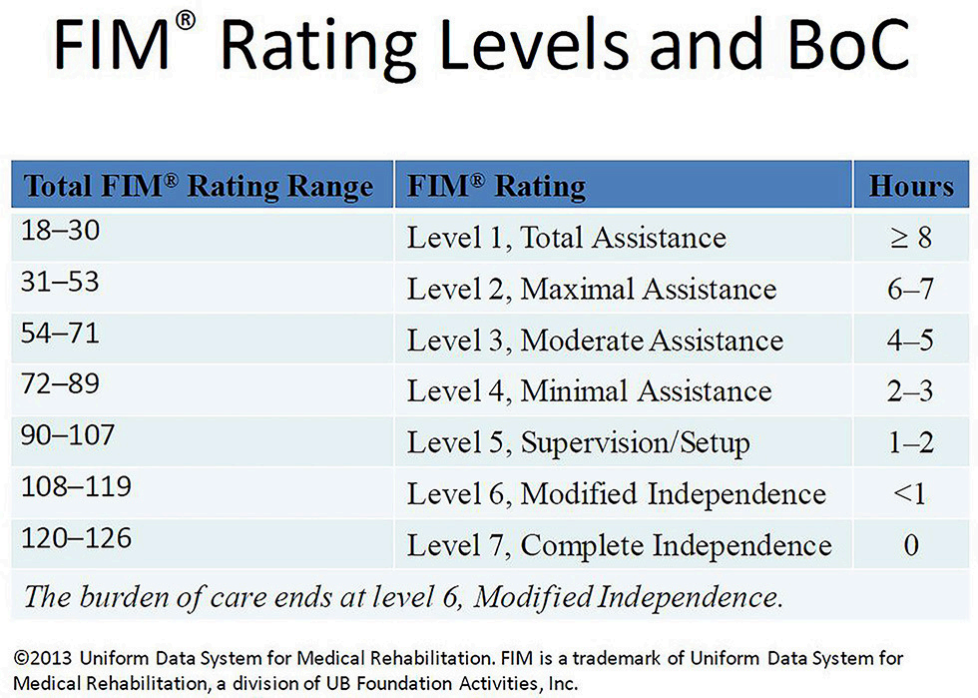
FIM rating levels and burden of care (BoC). This figure is a slide presented by Paulette Niewczyk, MPH, PhD, Director of Research, at UDSMR's 2013 Annual Conference in Orlando, Florida (Permission to reuse this slide was obtained from UDSMR in 2018).

FIM scores reflect the assistance required for patients to perform both basic and instrumental activities of daily living also defined as the burden of care (22). The FIM instrument assesses 18 functions (13 motor and 5 cognitive). The FIM motor subscale includes feeding, grooming, bathing, dressing the upper and lower body, toileting, bladder and bowel management, bed to chair or wheelchair transfers, toilet transfers, shower or tub transfers, locomotion (walking or wheelchair), and locomotion (stairs), while the FIM cognitive subscale constitutes comprehension, expression, social interaction, problem solving, and memory. Each individual function is rated from one (total assistance) to seven (complete independence). The total FIM score (sum of FIM cognitive and FIM motor scores) ranges from 18 (total dependence) to 126 (total independence). The FIM total score can be classified as <72: severe, 72–108: moderate, and 109–126: mild (27); or based on the information from Uniform Data System for Medical Rehabilitation (22), FIM total 18–30: level 1 (total assistance; requires ≥8 h of care in a 24 h period), 31–53: level 2 (maximal assistance; requires 6–7 h of care in a 24 h period), 54–71: level 3 (moderate assistance, requires 4–5 h of care in a 24 h period), 72–89: level 4 (minimal assistance, 2–3 h of care in a 24 h period), 90–107: level 5 (supervision or setup, 1–2 h of care in a 24 h period), 108–119: level 6 (modified independence, <1 h of care in a 24 h period), and 120–126: level 7 (complete independence, 0 h of care in a 24 h period; Figure 2).

#### Post-traumatic Amnesia (PTA)

This study utilizes the classification of extremely severe PTA (PTA >28 days) provided by the Handbook of Medical Neuropsychology (28) and TBIMS (24). In our study, individuals with a PTA duration ≤28 days were included in the non-extremely severe PTA (non-esPTA) group, while those with PTA duration >28 days were included in the extremely severe PTA (esPTA) group.

PTA can be evaluated in several ways (29, 30). In this study, the Orientation-Log (O-Log) was primarily used, and Galveston Orientation and Amnesia Test (GOAT) was occasionally used to evaluate PTA status. The O-Log is based on appropriate responses to questions regarding date, time, city, kind of place, name of hospital, month, date, year, day of week, clock time, etiology/event, and pathology deficits. O-Log has a maximum score of 30 (31, 32). Similar to O-Log, GOAT assesses a patient's orientation based on the responses to questions about people, location, date, time, and event with a total score of 100 (9, 33). O-Log or GOAT is performed each day as patient demonstrates clinical improvements. PTA emergence is defined as the first day when a patient obtains two consecutive scores of ≥25 on the O-Log or a score ≥76 on the GOAT.

#### Intracranial Pressure (ICP)

ICP was measured during acute hospital stay (if clinically indicated) using a monitoring device placed inside the skull. ICP values were abstracted from participants' medical records and entered into the TBIMS database as one of four following categories: ICP is <20 mmHg, ICP fluctuations are evident where peaks of ≥20 mmHg occur within one 24 h span, ICP fluctuations are evident where peaks of ≥20 mmHg occur over more than a 24 h span, and ICP remains ≥20 mmHg continuously for >24 h period. For this study, intracranial hypertension (ICH) is defined as the presence of ICP ≥20 mmHg (TBIMS ICP categories 2–4), which is in line with established clinical guidelines (34, 35). When assigning categories, coders made best efforts to exclude any transient spikes related to surgery, patient positioning, level of agitation, and other conditions, such as movement artifacts, that may artificially influence to the ICP measurement.

### Study Design

This study follows a retrospective longitudinal study design. Hypothesis was established after data collection from a prospective cohort study.

### Statistical Analysis

#### Participant Demographics

A total of 584 participants enrolled into TBIMS at SCVMC were compared to our final sample (*n* = 342) to test for the representativeness of our sample on age, sex, and race. Demographic information was further examined to compare esPTA and non-esPTA groups. A *t*-test was used to compare the average age, and chi-square tests were used to compare the distribution of sex and race.

#### Effects of esPTA on FIM Total Scores From Rehabilitation Admission to 5 Years Post-injury

The sample (*n* = 342) was divided into two subgroups based on PTA duration >28 days (esPTA: *n* = 185) or ≤28 days (non-esPTA: *n* = 157). A two group (es-PTA and non-esPTA) x five time point (rehabilitation admission, rehabilitation discharge, 1-, 2-, and 5-year post-injury) mixed model repeated measure ANOVA was used to assess within group and between group differences in FIM total scores at each time point. Significant main effects of Group, Time, and the Time x Group interaction were further explored using *post hoc* tests.

#### Subgroup Analysis: Associations Between ICH and esPTA

Not all subjects included in the PTA duration and FIM analysis had ICP measurements. Participants that did not receive ICP monitoring were excluded from the subgroup analyses involving ICP. To examine the association between ICH and esPTA, the group was divided into two sub-groups based on ICP. ICH group includes participants with ICP ≥20 mmHg (*n* = 89), and non-ICH group includes participants with ICP <20 mmHg (*n* = 121). The odds ratio test was employed to examine the relationship between these two factors.

## Results

### Participant Demographics

Sex, age, and race were examined to determine representativeness of the present sample (*n* = 342) with the larger TBIMS sample (*n* = 584), and no statistically significant difference was found with *p* = 0.32, *p* = 0.18, and *p* = 0.15, respectively.

Of the 342 individuals included in this study, the average age was 32 years old (15.8 SD); 72.2% of the participants were male and 43.3% were of an ethnic minority (predominately Hispanic or Asian). Fifty-four percent of the participants experienced PTA for more than 28 days (esPTA). The two PTA groups (esPTA and non-esPTA) were balanced on sex [$\chi_{\left(1\right)}^{2}=$ 2.4, *p* = 0.12], age [*t*_(340)_ = −0.50, *p* = 0.61], and race [$\chi_{\left(1\right)}^{2}$ = 0.80, *p* = 0.37] (Table 1).

**Table 1:** Participant demographics.

**Characteristic**		**Whole sample (*n* = 342)**	**Non-esPTA group** **(*n* = 157)**	**esPTA group** **(*n* = 185)**	***p*-values**
Sex (%)	Male	72.2	68	76	0.12
	Female	27.8	32	24	
Age	Mean (SD)	32 (15.8)	32 (16.3)	33 (15.4)	0.61
Age group (%)	16–25	49.4	53	46	
	26–35	15.2	11	19	
	36–45	13.5	14	13	
	46–55	10.2	8	12	
	56–65	6.7	8	6	
	≥66	5	6	4	
Race (%)	White	56.7	54	59	0.37
	Non-white	43.3	46	41	
	Black	4.1	3	5	
	Asian	9.6	12	8	
	Hispanic	26.3	27	25	
	Other	3.2	4	3	
PTA duration (%)	>28 days	54.1			
	≤28 days	45.9			
	<1 day	2.9			
	1–7 days	5.6			
	8–28 days	37.4			
ICP (#)	<20 mmHg	121	60	61	
	≥20 mmHg	89	22	67	

*Demographics table represents the distribution of age, sex, race, and post-traumatic amnesia (PTA) duration characteristics of the participants in the study. Mean (± SD) has value as years old. ICP is a subgroup that included only patients who had ICP monitoring (n = 210)*.

### Association Between PTA Duration and FIM Scores

**Figure 3:**
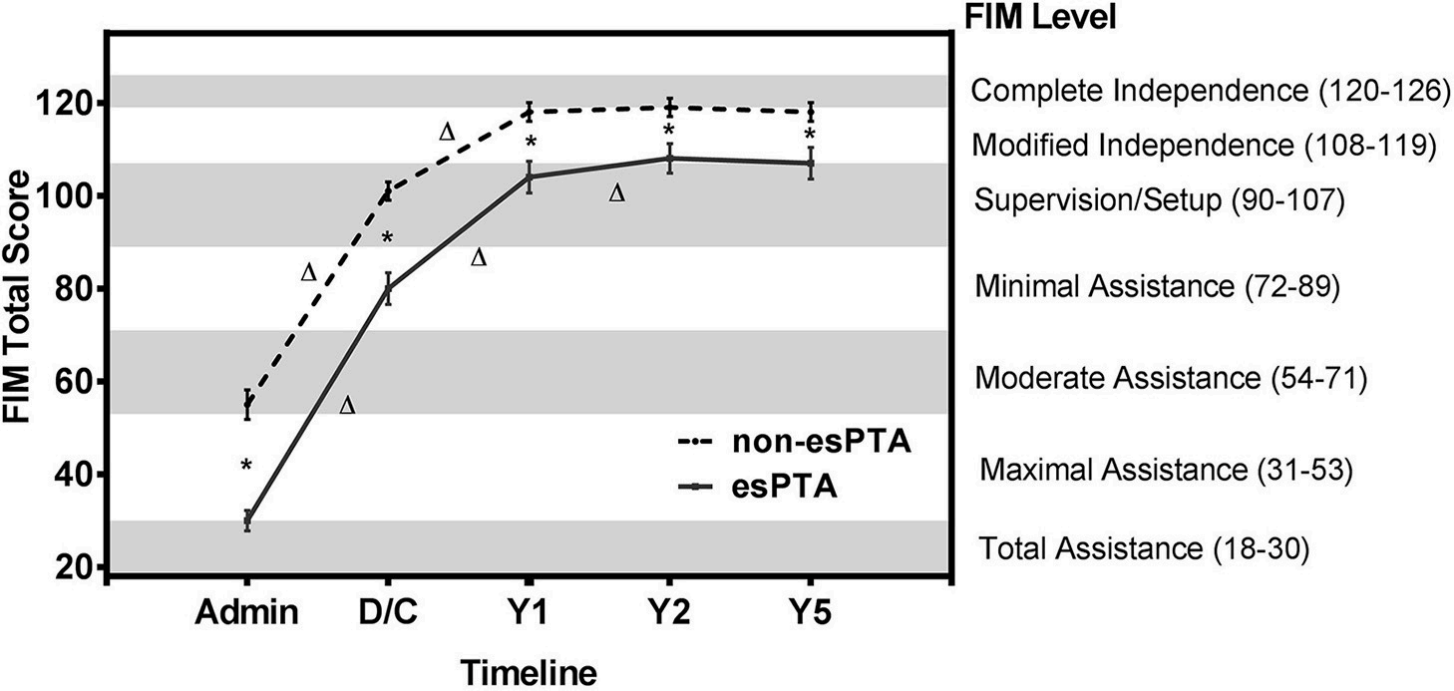
FIM total scores of esPTA group and non-esPTA group at 5 time points. Line plot illustrates the change in FIM total scores across follow-up periods in the esPTA and non-esPTA groups. The non-esPTA and esPTA groups's FIM scores are plotted with FIM total scores on the left y-axis and the corresponding seven FIM levels on the right y-axis with the timeline on the x-axis.^*^Represents a significant (*p* < 0.05) difference between groups at a single time point, and Δ represents a significant (*p* < 0.05) within group difference between time points. Error bars with 95% confidence intervals.

The non-esPTA group had higher FIM total scores across all time points (Figure 3 and Table 2). Mixed model repeated measure ANOVA for the 2 groups (esPTA vs. non-esPTA) x 5 time points (admission, discharge, year 1, 2, and 5) on the FIM total score reveals a main effect of group [*F*_(1, 340)_ = 115, *p* < 0.001, η^2^ = 0.25] and time [*F*_(2.3, 783.3)_ = 1,934, *p* < 0.001, η^2^ = 0.85] as well as a group x time interaction [*F*_(2.3, 783.3)_ = 18, *p* < 0.001, η^2^ = 0.05]. Both the main effect of time and group x time interaction were corrected using the Greenhouse-Geisser correction to account for the sphericity violation. *Post hoc* pairwise comparisons were used to further investigate the group x time interaction.

**Table 2:** esPTA group and non-esPTA group's FIM scores.

	**Non-esPTA Group** **(within group comparision)**			**esPTA Group** **(within group comparision)**			**Between groups**	
**Time**	**Mean**	**SD**	***p***	**Mean**	**SD**	***p***	***p***	**Eta^**2**^**
Admission	54.9	19.5		30.2	14.6		<0.001	0.35
Discharge	100.6	12.2	<0.001	80.3	23.6	<0.001	<0.001	0.22
Year 1	118.1	9.8	<0.001	103.8	23.7	<0.001	<0.001	0.13
Year 2	119.2	9.4	0.078	107.5	21.2	<0.001	<0.001	0.11
Year 5	118.3	13	0.274	107.2	22.5	0.667	<0.001	0.08

*This table summarizes the results for the repeated measures ANOVA. Year 1, 1-year post-injury follow-up; Year 2, 2-year post-injury follow-up; Year 5, 5-year post-injury follow-up*.

Tests of between-subjects effects revealed a significant higher FIM total score (greater independence) in the non-esPTA group compared to the esPTA group across all time points (*p* < 0.001; Figure 3 and Table 2). Within group comparisons across time revealed significant improvements in FIM total score in the non-esPTA group from admission to discharge and discharge to 1 year follow-up (*p* < 0.001) and no significant differences were observed from year 1 to year 2 (*p* = 0.078) and from year 2 to year 5 follow-up (*p* = 0.274) (Figure 3 and Table 2). The esPTA group showed significant improvements in FIM total score from admission to discharge, discharge to year 1, and year 1 to year 2 (*p* < 0.001); year 2 to year 5 did not show significant improvements (*p* = 0.667) (Figure 3 and Table 2).

### Subgroup Analysis: Associations Between ICH and esPTA

Out of 89 participants who experienced ICH, two thirds had esPTA (*n* = 67) compared to approximately half of the non-ICH group who presented with esPTA (61/121). Individuals in the ICH group were nearly three times more likely to have esPTA compared to individuals without ICH (Odds Ratio = 2.996, 95% CI: 1.645–5.453, *p* < 0.001, V = 0.252).

## Discussion

This study adds to the literature supporting the hypothesis that prolonged PTA duration significantly increases the burden of care (10–13, 36, 37) and this burden of care continues to affect patients up to 5 years post-injury. Individuals with esPTA show lower FIM total scores at each time point from rehabilitation admission to 5 years post-injury compared to those without esPTA. Based on the Uniform Data System's classification of FIM scores (Figure 2), the results of this study reveal individuals with esPTA on average require approximately twice as many hours of care across assessment periods compared to non-esPTA individuals. This increased burden of care is represented by either a 2 FIM level (admission) or 1 FIM level difference (discharge to 5-year follow-up) (Figure 3 and Table 2). At admission into an acute rehabilitation center, the esPTA group required ≥8 h of care, while the non-esPTA group required 4–5 h of care. The increased burden of care continued at discharge with the esPTA group requiring 2–3 h of care compared to the non-esPTA group requiring 1–2 h of care. At year 1, the esPTA group required 1–2 h of care, while the non-esPTA group required <1 h of care. At year 2 and year 5, the esPTA group was approaching Modified Independence translating to a requirement of <1 h of care, while the non-esPTA group approached Complete Independence translating to no requirement of care. These results demonstrate that differences in the burden of care remain present at 5 years post-injury, extending prior research where patients with PTA for <30 days are more than three times as likely to be living independently 1 year after injury compared to those with PTA extending more than 30 days (11). The results presented in this study have implications for long-term discharge planning of patients from acute rehabilitation as those with esPTA clearly have a greater long-term burden of care. In addition, unlike the non-esPTA group whose FIM total score plateaued 1 year post-injury, the FIM total score continued to improve up to 2 years post-injury for the esPTA group. The result demonstrates that esPTA individuals continue to make significant improvements and may benefit from continued therapeutic interventions, in order to maximize their recovery.

While there is a well-established literature base for the prognostic role of PTA duration, there have been limited studies to identify predictors of PTA duration. Sherer et al. used age, years of education, year of injury, GCS, length of coma, pupillary responsiveness, and intracranial operations to predict length of PTA (18). The important role of acute rehabilitation on PTA duration was highlighted by Saneda and Corrigan, as their data revealed that the time from injury to acute rehabilitation was a prognostic indicator for resolution of PTA (38). Interestingly, the Canadian North Star project found that patients who participated in an orientation program emerged from PTA 5 days earlier than the control group; however, this difference was not statistically significant (39). In our subgroup analysis for patients that received ICP monitoring, the results indicate that the likelihood of participants experiencing esPTA increases three times in participants with ICH. Prevention and treatment of ICH and secondary brain insults are emphasized in current critical care management guidelines for severe TBI (40). Efforts have been taken to reduce ICH in patients suffering from TBI because ICH is associated with poor outcomes (19–21). Elucidating the impact of ICH on PTA duration may benefit individuals with TBI in regard to planning for acute and long-term rehabilitation strategies. The results of this study show the association of ICH and PTA duration as well as support the implementation of additional studies to investigate the relationship between ICH and prolonged PTA from a structural standpoint and to investigate the implications of ICH treatment on PTA duration.

Several limitations should be considered when interpreting the results from this study. Retrospective studies are limited by the nature of data available. Although the variables collected were part of the TBIMS prospective longitudinal study that provides training and certification for data abstraction, data on acute care before rehabilitation admission was collected based on chart review. Certain variables like ICH treatment and absolute PTA duration of participants who were discharged in PTA were not available due to the nature of the TBIMS data collection. In order to include participants who were still experiencing PTA at discharge, our study used a literature based categorical scale for PTA duration. This highlights the importance of future studies accurately tracking PTA duration after discharge. Additionally, the sample included only participants admitted to a rehabilitation center with moderate to severe TBI, which may affect the application of this result to other TBI populations. Furthermore, while an investigation of the relationship between ICH and functional outcomes would be an interesting next step, the available data from the current retrospective study would not be sufficient to address this question. The TBIMS National Database focuses on data acquired from acute rehabilitation admission through post-discharge. In order to adequately investigate outcomes associated with ICH, a study with additional data focused on the acute stage of treatment would be needed. Another important limitation to the findings of the study includes the attrition rate of participants. However, given the long term follow up needed for this study, the sample size is reasonable and in line with reports of other centers participating in the TBIMS National Database (24, 41).

In conclusion, this study suggests that individuals with esPTA after TBI have a greater early and sustained burden of care which requires approximately two times as many hours of care from rehabilitation admission to 5 years post-injury compared to individuals without esPTA. Our data revealed that recovery plateaus at 2 years post-injury for individuals with esPTA, while recovery plateaus 1 year earlier for non-esPTA individuals. The study also shows that the likelihood of patients experiencing esPTA increases with ICH. These findings may help with prognostic and planning for patient care in the acute care setting as well as after being discharged from the hospital.

## Ethics Statement

This study was carried out in accordance with the recommendations of TBIMS ND standard operation procedures (https://www.tbindsc.org/SOP.aspx) with written informed consent from all subjects. All subjects gave written informed consent in accordance with the Declaration of Helsinki. The protocol was approved by the Santa Clara Valley Medical Center Institutional Review Board.

## Author Contributions

TD is the last author, directed the project, assisted in data interpretation and editing of the manuscript. NQ, RE, and BD analyzed data and wrote most of the article. All remaining authors contributed to both data interpretation and writing of the article.

### Conflict of Interest Statement

The authors declare that the research was conducted in the absence of any commercial or financial relationships that could be construed as a potential conflict of interest.
